# Identification of Five Developmental Processes during Chondrogenic Differentiation of Embryonic Stem Cells

**DOI:** 10.1371/journal.pone.0010998

**Published:** 2010-06-07

**Authors:** Akihiro Yamashita, Sandi Nishikawa, Derrick E. Rancourt

**Affiliations:** Department of Biochemistry and Molecular Biology, University of Calgary, Calgary, Alberta, Canada; University of Western Ontario, Canada

## Abstract

**Background:**

Chondrogenesis is the complex process that leads to the establishment of cartilage and bone formation. Due to their ability to differentiate *in vitro* and mimic development, embryonic stem cells (ESCs) show great potential for investigating developmental processes. In this study, we used chondrogenic differentiation of ESCs as a model to analyze morphogenetic events during chondrogenesis.

**Methodology/Principal Findings:**

ESCs were differentiated into the chondrocyte lineage, forming small cartilaginous aggregates in suspension. Differentiated ESCs showed that chondrogenesis was typically characterized by five overlapping stages. During the first stage, cell condensation and aggregate formation was observed. The second stage was characterized by differentiation into chondrocytes and fibril scaffold formation within spherical aggregates. Deposition of cartilaginous extracellular matrix and cartilage formation were hallmarks of the third stage. Apoptosis of chondrocytes, hypertrophy and/or degradation of cartilage occurred during the fourth stage. Finally, during the fifth stage, bone replacement with membranous calcified tissues took place.

**Conclusions/Significance:**

We demonstrate that ESCs show the chondrogenic differentiation pathway from the pluripotent stem cell to terminal skeletogenesis through these five stages *in vitro*. During each stage, morphological changes acquired in preceding stages played an important role in further development as a scaffold or template in subsequent stages. The study of chondrogenesis via ESC differentiation may be informative to our further understanding of skeletal growth and regeneration.

## Introduction

Cartilage is the first skeletal tissue to be formed during embryogenesis and participates in endochondral or perichondral ossification within developing, growing and regenerating bones [1]. During skeletal development, chondrogenesis and subsequent bone formation is a multi-step process that is tightly regulated by dynamic morphogenetic events and phenotypic changes [2]. During chondrogenesis, chondrocytes proliferate, maturate and ultimately undergo hypertrophy and apoptosis [1], [2]. A better understanding of the natural developmental process of chondrogenesis would help to create more efficient cartilage and bone therapeutic modalities.

Many efforts have been explored for understanding the morphogenetic event of chondrogenesis in normal individuals [1]. Due to their ability to differentiate into all cell types in the body, embryonic stem cells (ESCs) show great potential for investigating and understanding the developmental processes that occur during organogenesis, including cartilage and bone formation [3]. Therefore, we hypothesized that an ESC differentiation model system would offer the possibility of following this multi-step process from progenitor cells to terminal development. The differentiation of ESCs would provide an attractive model for further understanding skeletal development and regeneration.

We and others have previously explored ESC chondrogenesis *in vitro* [3]–[8]. Although the cellular events were described well in these studies, they had not examined the multi-step process of morphogenetic events and phenotypic changes using the ESC-derived cartilaginous aggregates by histomorphometry and immnohistochemistry in detail. In addition, even though endochondral ossification surrounding the connective tissue was shown *in vivo*, *in vitro* bone replacement through cartilage degradation without vasculogenesis remained unclear [9], [10].

In this study, based on our previous report of the direct chondrogenic differentiation of ESCs via the micro-mass culture approach, we examined the long-term expansion of cartilaginous aggregates in static suspension culture. Here, we demonstrate the multi-step developmental processes of chondrogenesis and subsequent bone replacement *in vitro*. Accordingly, we define the five main stages of chondrogenesis by the cellular, extracellular and molecular changes occurring in ESC chondrogenic differentiation.

## Materials and Methods

### ESC chondrogenic differentiation

Mouse D3 ESCs (American Type Culture Collection, Rockville, Maryland, USA) were differentiated into the chondrocyte lineage using micro-mass culture. Dissociated ESCs were cultured at a high-density, 1.0×10^5^ cells per 10 µl×9 spots in 6 cm culture dish for 2 hours [8], [11]. After incubation, medium was added to each dish without dissociating the cell drops. For chondrocyte differentiation, we used differentiation media containing DMEM (Gibco), 1% non-essential amino acids (Invitrogen), 50 U/ml penicillin and 50 µg/ml streptomycin (Invitrogen), 0.1 mM 2-mercaptoethanol (Invitrogen), 1% ITS (Invitrogen), 1% FBS (Gibco), 10 ng/ml TGF-β1 (PeproTech), 10 ng/ml BMP-2 (PeproTech), and 50 µg/ml Ascorbic acid (Sigma). Medium was changed every 2 days.

**Figure 1:**
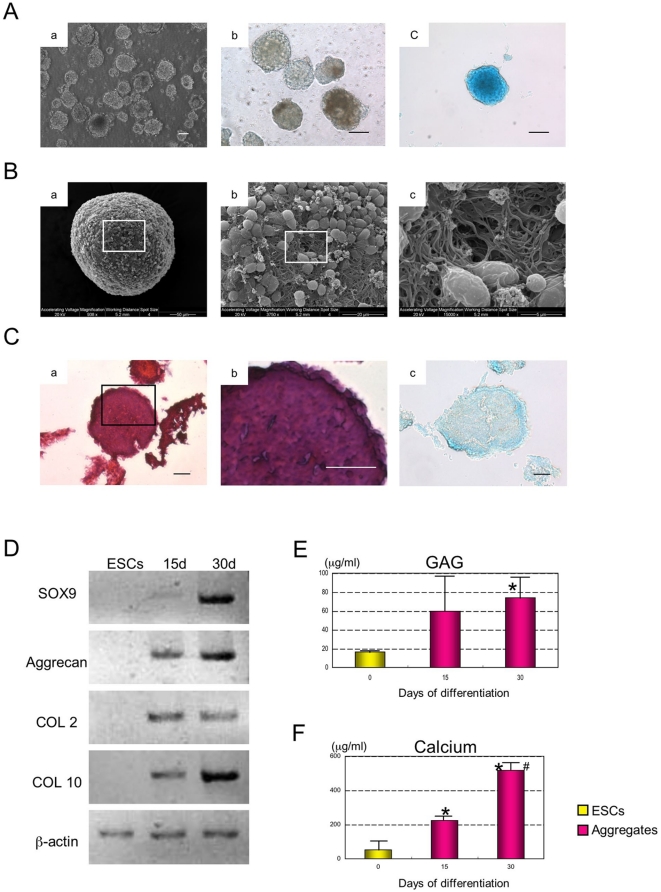
Condensation of differentiated ESCs. (A) The morphology of ESC chondrogenic aggregates cultured at day 5 (a) and 15 (b), Alcian Blue at day 15 (c). Scale bars represent 100 µm. (B) The ultra-structure of static suspension aggregates was analyzed by SEM on day 15 (a, b, c). (C) The H&E histological section of chondrogenic aggregate cultured at day 15 (a), Higher magnification (b), Alcian Blue (c). Scale bars represent 20 µm. (D) Expression of Sox9, Aggrecan, COL 2 and COL 10 mRNAs were analyzed by semi-quantitative RT-PCR at 0 (ESCs), 15 and 30 days of differentiation. β-actin was used as an internal control. (E) GAG content was determined. Data is expressed as mean ± SD (n = 4) per well. * Significant difference from day 0 (ESCs). P<0.05 with Student's *t*-test. (F) Calcium accumulation during differentiation was determined. Data is expressed as mean ± SD (n = 4) per well. * Significant difference from day 0 (ESCs). ^#^ Significant difference from day 15 P<0.05 with Student's *t*-test.

Differentiated ESCs formed aggregates (Fig. 1Aa). After 5 days, these aggregates were separated from the dishes by pipetting and transferred to suspension culture in 6 cm petri dishes containing differentiation media containing DMEM, 1% non-essential amino acids, penicillin streptomycin, 0.1 mM 2-mercaptoethanol, 1% ITS, 1% FBS, 10 ng/ml BMP-2 and 50 µg/ml Ascorbic acid.

### Scanning electron microscopy

Aggregates were sampled at day 15 and 40. These specimens were fixed in 2.5% gluteraldehyde (Sigma) in 1M Sodium Cacodylate buffer (Sigma) for 1 hour. After washing with Sodium Cacodylate buffer, samples were fixed by 2% Osmium (Electron Microscopy Sciences) for 15 min. The samples were dehydrated in increasing percentages of anhydrous ethyl alcohol (50%, 75%, 90% and 100%). The samples were dried using increasing percentages of Hexamethyl disilazine (HMDS, Sigma) diluted in anhydrous ethyl alcohol(10%, 30%, 50%, 70%, 80%, 90% and 100%). After air drying overnight, aggregates were coated with gold using a Hummer 1 Sputter Coater. The samples were examined by SEM (Phillips/FEI ESEM XL-30) operated at working distance of 5.2–10.7 mm and an accelerating voltage of 20 kV [11].

### GAG assay

To determine the total glycosaminoglycan (GAG) from one micro-mass containing 20–30 aggregates samples were taken at day 15 and 30 of differentiation. The cultures were digested with 100 µl of papain digestion buffer (4.5 unit/ml in 50mM Phosphate buffer, pH6.5, with 2mM N-acetylcysteine and 2mM Na_2_EDTA, Sigma) for 4 hours at 65°C. Values are calculated from a standard curve using Chondroitin Sulfate-A Sodium Salt (Sigma), which was measured along with the samples at 510 nm in a Benchmark Plus microplate spectrophotometer (BioRad) [8].

### Calcium Assay

To measure calcium accumulation from one micro-mass containing 20–30 aggregates samples were taken at day 15 and 30 of differentiation. The cultures were digested with 100 µl of 10% formic acid for 4 hours room temperature. Values were calculated from a standard curve using Arsenazo III (DCL), which was measured along with the samples at 650 nm in a Benchmark Plus microplate spectrophotometer according to the manufacturer's instructions [8].

### RNA isolation and semi-quantitative and quantitative RT-PCR

Total RNA was isolated from cell culture at day 0, 10, 15, 20, 30, 40, 50 and 60 of differentiation using the RNeasy Mini Kit (Qiagen) according to the manufacturer's instructions with on-column DNase I digestion. The amount of total RNA was measured using a Bio Photometer (Eppendorf). One microgram of total RNA was used as a template for cDNA synthesis with the Super Script III First-Strand Synthesis System (Invitrogen). The PCR reactions (20 µl) were carried out using Taq DNA Polymerase (Invitrogen) according to the manufacturer's instructions. PCR conditions were: 3 min at 94°C, 30 sec denaturation at 94°C, 45 sec annealing at 55°C, and 1 min 30 sec extension at 72°C. Primers were designed based on the mouse sequence and BLASTed for their specificity at the National Center for Biotechnology Information (NCBI) [12]. Primer sequences are described in Table 1.

**Table 1:** Primer Sequences.

Gene	Primer
Sox9	For: 5′-AGCTCACCAGACCCTGAGAA-3′
	Rev: 5′-GATTCTCCAATCGTCCTCCA-3′
Aggrecan	For: 5′-CAGGGTTCCCAGTGTTCAGT-3′
	Rev: 5′-CTGCTCCCAGTCTCAACTCC-3′
COL1	For: 5′-CTGCCTGCTTCGTGTAAA-3′
	Rev: 5′-ACGTTCAGTTGGTCAAAGGTA-3′
COL2	For: 5′-CCGTCATCGAGTACCGATCA-3′
	Rev: 5′-CAGGTCAGGTCAGCCATTCA-3′
COL10:	For: 5′-AAGGAGTGCCTGGACACAAT-3′
	Rev: 5′-GTCGTAATGCTGCTGCCTAT-3′
MMP13	For: 5′-CAGTTGACAGGCTCCGAGAA-3′
	Rev: 5′-CGTGTGCCAGAAGACCAGAA-3′
Cbfa1	For: 5′-CCGCACGACAACCGCACCAT-3′
	Rev: 5′-CGCTCCGGCCCACAAATCTC-3′
Osteocalcin	For: 5′-TCTCTCTGCTCACTCTGCTGG-3′
	Rev: 5′-ACCGTAGATGCGTTTGTAGGC-3′
β-actin	For: 5′-GGCCCAGAGCAAGAGAGGTATCC-3′
	Rev: 5′-ACGCACGATTTCCCTCTCAGC-3′

Amplified products were used to derive standard curves for quantitative (real-time) RT-PCR. Real-time PCR was performed in an iCycler iQ system using a SYBR green PCR master mix (Biorad), with the following cycle conditions: an initial denaturation step of 95°C for 3 min was performed, followed by 45 cycles of denaturation at 95°C for 30 sec, annealing at 57°C for 30 sec and extension at 72°C for 30 sec [13].

### *In vitro* aggregate assay

Aggregates were sampled at day 15, 30, 40, 50, 60 and 100. These specimens were fixed in 4% paraformaldehyde. After dehydration in ascending concentrations of ethanol and xylene, the specimens were embedded in paraffin. The paraffin sections were then deparaffinized, hydrated, and stained with hematoxylin and eosin (H&E), Alcian Blue (Wako), Alizarin Red S (Wako), Methylene Blue, COL 1 and COL 2 (Santa Cruz). Methylene Blue consisted of methylene blue (Sigma) and 0.03% basic fuchsin (Sigma) [9], [11]. Methylene Blue with basic fuchsin binds to collagen [9].

### *In vivo* implant assay

To determine chondrogenic potential *in vivo*, 20–30 aggregates were injected into the femoral muscle of SCID mice, using a 25 gauge syringe. ESCs were differentiated into chondrocyte lineage *in vitro* for 30 days; these aggregates were then transplanted into the SCID mice for another 4 weeks of growth. SCID mice were obtained from Taconic and housed in the single-barrier animal facility of the Faculty of Medicine, University of Calgary [8]. After 4 weeks the animals were sacrificed and tissue at the point of injection was dissected. These specimens were fixed in 4% paraformaldehyde and decalcified in 10% EDTA (pH 8.0) solution for 1 week. After dehydration in ascending concentrations of ethanol and xylene, the specimens were embedded in paraffin. The paraffin sections were then deparaffinized, hydrated, and stained with H&E, Toluidine Blue (Wako), and Methylene Blue.

### Immunofluorescence

Cells were fixed in 4% paraformaldehyde for 15 min and washed twice with PBS-T. After permeabilization with PBS containing 0.1% Triton-X, cells were washed twice with PBS-T, blocked with PBS containing 3% BSA for 1 hour at room temperature, and incubated with primary antibody (Santa Cruz, 1∶200 with 3%BSA/PBS) overnight at 4°C. The cells were washed three times with PBS-T followed by incubation with Alexa flour 488 conjugated secondary antibody (1∶500 with PBS) and DAPI (1∶500 with PBS) for 2 hours at room temperature. Unbound secondary antibodies were removed by three washes with PBS-T. Fluorescent images were captured using a fluorescent microscope (IX70, Olympus, Japan) equipped with CCD camera (RT Color, Diagnostic, Spot Software V4.0.9) [8].

### TUNEL staining

To identify the apoptotic cells, TUNEL staining was performed using the *in situ* cell apoptosis detection kit according to the manufacturer's instructions (Roche). Briefly, sections were fixed with 4% paraformaldehyde for 30 min. Terminal deoxynucleotidyl transferase (TdT) and biotin-11-dUTP reactions were performed for 1 hour at 37°C. Fluorescent images were captured using a fluorescent microscope equipped with CCD camera [8].

### Statistical analysis

Means ± S.E.M. were calculated and statistically significant differences between two groups were determined using the Student's *t*-test at *P*<0.05 (Excel, Microsoft).

## Results

### Cell condensation and spontaneous scaffold formation

Following micro-mass chondrogenic differentiation, the resulting aggregates were cultured in static suspension (Fig. 1Aa). At day 15, these spherical aggregates showed Alcian Blue positive staining, indicating the presence of proteoglycans in the aggregates (Fig. 1Ab, c). Using SEM imagining, aggregates were found to consist of many round-shaped cells with some fibrils (Fig. 1B). The extending fibers formed networks and had the appearance of a fibril scaffold (Fig. 1Bc). Sectioning of the aggregates revealed cells that were tightly connected by these fibrils, resulting in uniform and compact cell aggregate formation (Fig. 1C). Pores were not observed during this phase of chondrogenic differentiation. These results indicated that chondrogenic differentiation of ESCs formed uniform and compact cell aggregates with the network of fibrils by day 15.

### Chondrocyte differentiation and its plasticity in adhesion culture

**Figure 2:**
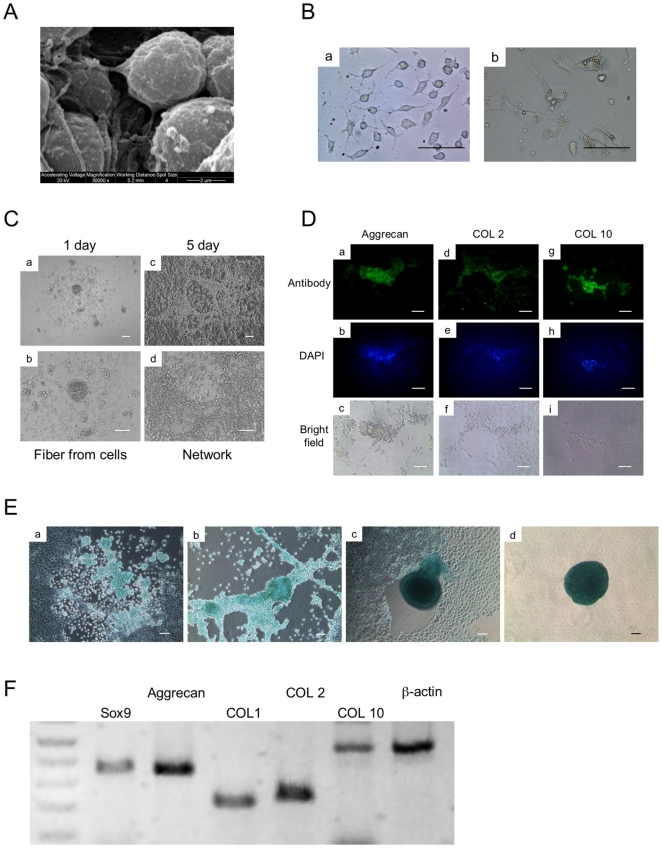
Chondrocyte differentiation. (A) The ultra-structure of static suspension aggregate was analyzed by SEM at day 15. (B) The cells shed from aggregates displayed two types, round-shaped with fibrils (a) and flat fibroblastic cells (b). Scale bars represent 20 µm. (C) The round-shaped cells formed network spontaneously. Scale bars represent 50 µm. (D) The expression of chondrocyte-related proteins (Aggrecan, COL 2, COL 10) was analyzed at day 20 of differentiation by immunofluorescence (a, d, g), DAPI (b, e, h), Bright field (c, f, i). Scale bars represent 50 µm. (E) Alcian Blue stained chondrogenic cells at day 20 of differentiation (a, b). These cells were induced to form the aggregates spontaneously (c, d). Scale bars represent 50 µm. (F) The expression of chondrocyte-related mRNAs (Sox9, Aggrecan, COL 2, COL 10) and fibroblast-related mRNA (COL 1) were analyzed at day 20 by semi-quantitative RT-PCR. β-actin was used as a loading control.

In this study, aggregates were cultured in Petri dishes. These aggregates showed chondrogenic potential as indicated by PCR, GAG and calcium accumulation (Fig. 1D–F). A small population of cells was found to be sloughed off from the surface of the cell aggregate and attached to the Petri dish. Interestingly, both round-shaped and fibroblastic cells were observed (Fig. 2B). Round-shaped cells had similar fibrils to those seen in the suspension culture aggregates (Fig. 2A, Ba, C). These fibrils connected with each other, resulting in a network formation (Fig. 2C). These cells were found to express the chondrocyte-related genes, Aggrecan, collagen (COL) 2 and 10, and Sox9 based on semi-quantitative RT-PCR (Fig. 2F). Chondrocyte related proteins, Aggrecan, COL 2, and COL 10 and proteoglycans were also found to be present using immunoflourescence (Fig. 2D) and staining for Alcian Blue (Fig. 2E), respectively. These results indicated that the round-shaped cells shed from the aggregates were chondrocytes. As the culture process progresses, these chondrocytes spontaneously formed cartilaginous aggregates (Fig. 2E).

### Extracellular matrix deposition and cartilage formation

**Figure 3:**
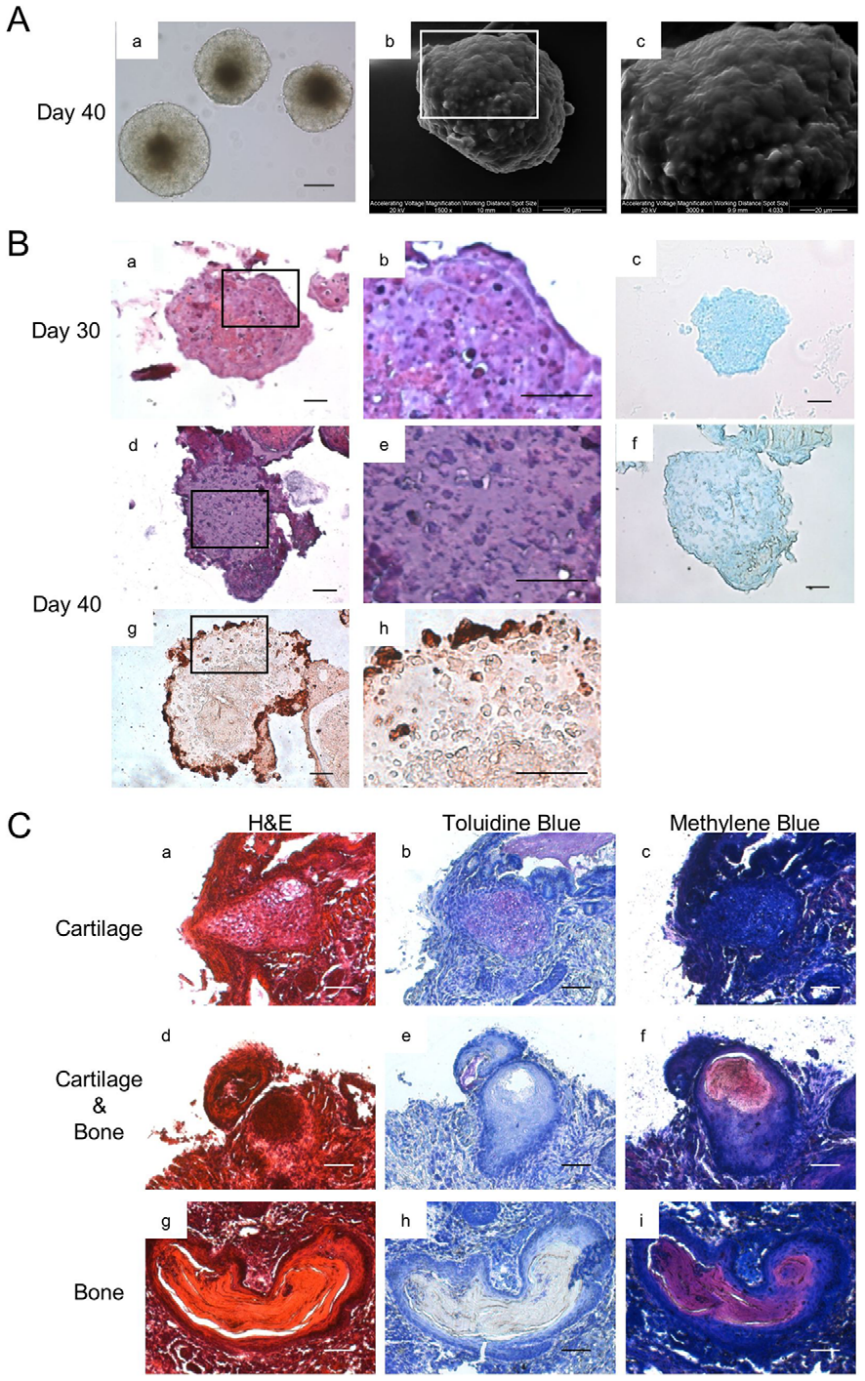
*In vitro* cartilage formation, and *In vivo* cartilage and bone formation. (A) The morphology and ultra-structure of aggregate cultured at day 40. Morphology (a), Scale bars represent 100 µm. SEM (b, c) (B) The histological section of ESC chondrogenic aggregate cultured at day 30 and 40, H&E staining (a, d). Higher magnification (b, e). Alcian Blue (c, f). COL 2 (g, h). Scale bars represent 20 µm. (C) Transplantation of aggregates. Cells were cultured *in vitro* for 30 days under the chondrogenic differentiation protocol. The aggregates were then transplanted into SCID mice. After removal of transplanted tissue, both cartilage and bone tissues were observed. Cartilage was indicated by Toluidine Blue (b, e). Bone was indicated by Methylene Blue (f, i). Scale bars represent 50 µm.

As the culture progressed, the margins of the aggregates became sharply defined (Fig. 3Aa). The fibrils observed previously on day 15 had disappeared, and were replaced by a smooth surface observed on day 40 (Fig. 3Ab, c). Sectioning of aggregates on day 30 showed that extracellular matrix (ECM) was deposited around the cells. An abundance of ECM surrounding the lacunae was also observed on day 40 (Fig. 3B). The aggregates appeared to have morphology similar to that found in cartilage (Fig. 3Be). These results indicated that the cells in the aggregates change their morphology and the fibrils were replaced with cartilaginous ECM during long term differentiation.

Following transplantation, the aggregates formed cartilage (Fig. 3Ca–f), as well as bone (Fig. 3Cd–i) *in vivo*. These results indicated that the aggregates had the potential to continue to proliferate and differentiate *in vivo* through chondrogenesis and chondral ossification.

### Hypertrophy and degradation of cartilage and subsequent bone replacement

**Figure 4:**
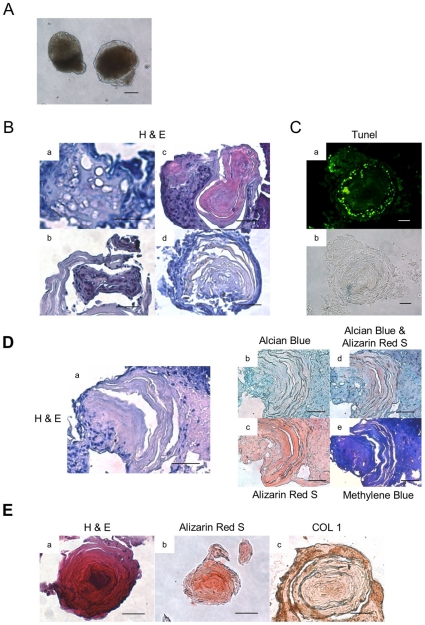
*In vitro* hypertrophy and degradation of cartilage, and bone replacement. (A) The morphology of aggregate cultured at day 50. Scale bars represent 100 µm. (B) The histological section of aggregate cultured at day 50 or 60 was stained by H&E. Hypertrophic cartilage (a), Degraded cartilage (b), Degraded cartilage and bone replacement (c) and bone replacement (d). Scale bars represent 20 µm. (C) Apoptotic cells were indicated by TUNEL staining. TUNEL (a), Bright field (b). Scale bars represent 20 µm. (D)The section of bone replacement cultured at day 60. H&E (a), Alcian Blue (b), Alizarin Red S (c), Alcian Blue & Alizarin Red S (d), Methylene Blue (e). Scale bars represent 20 µm. (E) The section of bone cultured at day 100. H&E (a), Alizarin Red S (b), COL 1 (c). Scale bars represent 20 µm.

During further development, a dark core appeared in the centre of aggregates by day 50 (Fig. 4A). Sectioning of these aggregates showed the presence of hypertrophic cartilage (Fig. 4Ba). Degraded cartilage was found surrounding membranous bone tissue (Fig. 4Bb, c, d). The degraded cartilage was also found to include apoptotic cells as indicated by TUNEL staining (Fig. 4C). During this phase of chondrogenesis, the amount of proteoglycans present is decreasing with a concomitant increase in calcification (Fig. 4D). Interestingly, high calcification was evident in the membranous tissues replacing the cartilage as indicated by Alcian Blue staining (Fig. 4Dd). In this way, cartilage was replaced by bone-like membranous tissue. More regions of bone were observed by day 100 (Fig. 4E). These results indicated that hypertrophic and degraded cartilages were formed, resulting in the aggregates forming calcified tissue resembling bone *in vitro* at a later stage.

### Gene expression patterns during ESC chondrogenesis in suspension

**Figure 5:**
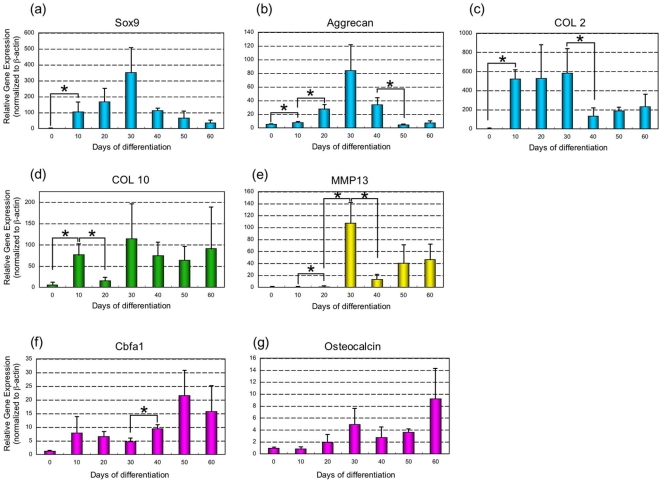
Gene expression during chondrogenesis in suspension culture. The expression of chondrocyte and osteoblast-related genes was analyzed from 0 to 60 days of differentiation by real-time RT-PCR. Sox9 (a), Aggrecan (b), COL 2 (c), COL 10 (d), MMP13 (e), Cbfa1 (f), Osteocalcin (g). Data is expressed as means ± SD (n = 3) per lane. With a P<0.05 using the Student's *t*-test, * represents a significant difference between two.

We next used real time RT-PCR to examine the expression of chondrocyte, osteoblast and other markers during suspension culture (Fig. 5). Sox9, a chondrocyte transcription factor, which induces the expression of cartilage matrix, was significantly up-regulated after chondrogenic induction and peaked at day 30 (Fig. 5a). Expression of the cartilage ECM protein marker, Aggrecan and COL 2 peaked by day 30 and decreased by day 40 (Fig. 5b, c). Based upon the expression of COL 10, hypertrophic chondrocytes appeared within the population as early as day 10. After down-regulation at day 20, COL 10 was up-regulated again by day 30 and remained at moderate levels throughout the course of the culture period (Fig. 5d). MMP13, a protease expressed by terminal hypertrophic chondrocytes, peaked at day 30 and up-regulated again at day 50 and 60 (Fig. 5e). Overall, the expression of chondrocyte-related genes was up-regulated by day 30.

Cbfa1 expressed by chondrocytes was maintained through terminal hypertrophic differentiation and induced bone formation, up-regulated during differentiation and peaking at day 50 and 60 (Fig. 5f). The expression of Osteocalcin a late marker of bone ECM peaked by day 60 (Fig. 5g).

## Discussion

The field of regenerative medicine promises the development of cell-based therapies [15]. ESCs may provide a source of these cells, due to their ability to provide large numbers of specialized cell types via *in vitro* differentiation [16], [17]. However, the greatest challenge facing the use of ESCs in regenerative medicine and tissue engineering is determining how cells respond and form tissue during differentiation and development. The successful use of ESC-derived cells for clinical application requires a pure cell population, maintenance of differentiated cell phenotype and constitution of functional tissue *in vitro* and *in vivo* [9], [10], [18]. Therefore, a better understanding of the developmental process would have a significant impact on the design of strategies for regenerative medicine using ESCs.

Chondrogenesis is the earliest phase of skeletal development. This involves condensation of progenitors, chondrocyte differentiation, and deposition of cartilaginous extracellular matrix (ECM), resulting in the formation of cartilage and bone during chondrogenesis [2]. In this study, we focused on understanding the morphogenetic events during chondrogenic differentiation of ESCs in static suspension culture. Each stage was characterized as an established *in vitro* ESC developmental process of chondrogenesis.

As we mentioned previously, the first step in chondrogensis is cell condensation and the subsequent formation of condensed cell aggregates that occurs prior to chondrogenic differentiation [19], [20]. At this stage, factors such as the TGF-β superfamily (TGF-β and BMPs) are known to play critical roles in the compaction of progenitors cells and shaping of the condensations [21]. The biochemical effect of growth factors in concert with physical factors such as this micro-mass approach and self-assembly potential of ESCs would promote spherical cell condensation. The combination of biochemical effects, culture environment and characteristic of ESCs helps to create cell condensations as the cellular modules or units from which skeletons are built.

The second stage of chondrogenesis has been associated with changes in cell morphology and concomitant changes in gene expression for cell fate commitment. During this stage, ESCs differentiated into chondrocytes within spherical aggregates. In particular, the expression of a transcriptional factor Sox9 induces progenitor cells to become chondrocytes during cell condensations [1], [2], [22]. In this study, differentiated ESCs were shown to have fibril networks, resulting in compact cell aggregates (Fig. 1B, C). The network of fibrils served as a scaffold for further cartilaginous deposition and cartilage formation. Under appropriate conditions, differentiated ESCs had the potential to develop the phenotypic features of the chondrocyte and create a suitable environment for cartilage formation by itself.

The round-shaped cells seen in the adherent cultures had similar characteristics of the chondrocyte such as a fibril network, expression of proteoglycans and other chondrocyte-related markers (Fig. 2). However, these cells showed plasticity of their phenotypes, as both round-shaped and fibroblastic cells were found in an adhesion culture (Fig. 2B). It has been previously reported that the phenotypic features of chondrocytes may change into fibroblastic cells morphologically as the culture process progresses on a two-dimensional substrate [14]. This morphological change has previously been associated with the loss of the chondrocytic phenotype- [14]. Therefore, in terms of not only producing a sufficient number of pure chondrocytes but also the suppression of cellular dedifferentiation, it is important that the spherical conformation be maintained in suspension culture. These findings suggest that the culture environment is an important factor in the regulation of differentiation of chondrocytes as well as stabilization of their phenotypes.

After expression of Sox9, chondrocytes synthesize cartilaginous ECM [1], [2], [22]. In this study, copious amounts of ECM were deposited, resulting in cartilage formation *in vitro* (Fig. 3B). Fibril networks that were formed previously were replaced by ECM and seemed to serve as a framework for cartilage formation. It is noteworthy that these aggregates showed chondrogenic activity, as cartilage formation and replacement with bone-like membranous tissues occurred *in vivo* (Fig. 3C). For this reason, this system would provide the opportunity not only to analyze the process of chondrogenesis but also to determine the *in vivo* responses of differentiated ESC when they are transplanted. This *in vivo* analysis could support our *in vitro* observations.

During development, terminally differentiated cells withdraw from the cell cycle, initiating the process of hypertrophic differentiation. At this stage, chondrocytes undergo hypertrophy and apoptosis to render hypertrophic cartilage and degradation, respectively [23], [24]. The process of chondral ossification occurs in the development of long bones from a cartilagenous anlagen [24], [25]. In this study, hypertrophic and degraded cartilages with bone-like membranous tissues were observed at 50 or 60 days (Fig. 4B, C). Their residual cartilage matrix seemed to serve as a scaffold for further calcium deposition and bone formation.

Finally, bone is deposited on remnants of the cartilage matrix through chondral ossification [9], [24]. In this study, calcified and bone-like membranous tissues were observed at late stages *in vitro* (Fig. 4D, E). Primary bone formation seemed to occur via cartilage resorption. In previous investigations of chondral ossification, calcification was observed within aggregates, it was thought to require the *in vivo* environment with vasculogenesis from the neighboring connective tissue [9], [10], [21], [24]–[26]. Interestingly, we could observe primary bone formation with bone-like membranous tissues via cartilage resorption *in vitro*. However, for more functional secondary bone formation including bone remodeling, it will require collaboration with the haematopoetic stem cell niche, such as vascular invasion *in vivo*, as has been previously reported [26].

The relative temporal aspects of each stage of chondrogenesis were also denoted by their associated molecular processes. After the first appearance of chondrogenic markers (Sox9, Aggrecan, COL 2), which decline thereafter, the hypertrophic chondrogenic marker (COL 10) appear almost immediately with sustained expression (Fig. 2D, 5). These cells seemed to commit to apoptosis at day 50, as shown by TUNEL staining (Fig. 4C). The expression of protease and osteogenic markers (MMP13 and Osteocalcin, respectively) resulted in degraded cartilage and primarily bone formation at day 50 and 60 (Fig. 4D).

**Figure 6:**
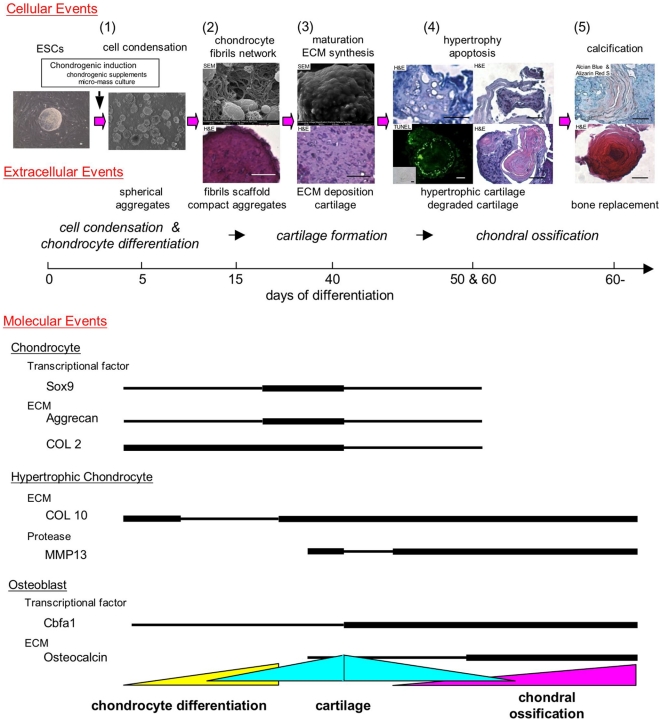
Schematic summary of the stages of chondrogenesis of ESCs. The relative temporal aspects of five stages of chondrogenesis are denoted by cellular, extracellular and molecular events: (1) Condensation of differentiated ESCs, (2) Differentiation into chondrocytes and fibril scaffold formation, (3) ECM deposition and cartilage formation, (4) Hypertrophy and degradation of cartilage, and (5) Bone replacement. Scale bars represent 20 µm.

Overall, we have demonstrated that ESCs exhibit dynamic morphological changes encompassing chondrogenic differentiation (Fig. 6). ESC chondrogenesis can be characterized by five main stages of cellular, extracellular and molecular events: (1) Condensation of differentiated ESCs, (2) Differentiation into chondrocytes and fibril scaffold formation, (3) ECM deposition and cartilage formation, (4) Hypertrophy and degradation of cartilage, and (5) Bone replacement. At each stage, morphological changes played an important role in further development as each stage acts as a scaffold or template for later stages. These processes, which would not be physically sequestered during development, were controlled in a temporal-spatial manner.

For clinical application, it will require the appropriate scales and timing of implants to fuse with the host tissues to recover natural shape safely [1]. A precise knowledge of how to control chondrogenesis would be essential for cartilage and bone tissue engineering. However, in the *in vivo* environment there are still unknown factors governing the differentiation of cells. The microenvironment of cells in the existing tissue, immunological responses and the hematopoetic niche all play important and complex roles in the regeneration process. Our observation of five overlapping stages during *in vitro* ESC chondrogenesis provides an opportunity to understand processes within a controlled environment *in vitro*. Accordingly, a better understanding of chondrogenesis should benefit skeletal regenerative medicine.
